# Retinopathy of Prematurity in Port Harcourt, Nigeria

**DOI:** 10.1155/2014/481527

**Published:** 2014-02-04

**Authors:** Adedayo O. Adio, Rosemary O. Ugwu, Chidi G. Nwokocha, Augusta U. Eneh

**Affiliations:** ^1^Department of Ophthalmology, University of Port Harcourt Teaching Hospital, Port Harcourt, Rivers State, Nigeria; ^2^Department of Paediatrics, University of Port Harcourt Teaching Hospital, Rivers State, Nigeria

## Abstract

*Purpose*. With many preterm babies now surviving as a result of improvement in neonatal care in Nigeria, the incidence of visual impairment/blindness as a result of retinopathy of prematurity (ROP) may rise. We describe our findings after screening starts for the first time in a 15-year-old special care baby unit so as to establish the incidence and risk factors for developing ROP. *Methods*. A prospective study carried out at the Special Care Baby Unit (SCBU) and Pediatric Outpatient Clinics of the University of Port Harcourt Teaching Hospital between January 1 and October 31, 2012. Fifty-three preterm babies (of 550 neonates admitted within the study period) delivered before 32 completed weeks and weighing less than 1500 g were included in the study following informed consent and the main outcome measure was the development of any stage of ROP. *Results*. Mean gestational age at birth was 28.98 ± 1.38 weeks. Mean birth weight was 1411 ± 128 g. Out of 550 babies admitted at SCBU, 87 of 100 preterms survived with 53 included in study. Twenty-five (47.2%) had different degrees of ROP with prevalence found to be 47.2%. Prevalence was higher (75%) in babies weighing <1300 g and those delivered before 30-week gestation (58%). Twenty-one (84%) had stage 1 no plus disease and 3 (12%) had stage 2 no plus disease. Only 1 (4%) had threshold disease in Zone 1. None had disease at stage 4 or 5 or AP-ROP. Receiving supplemental oxygen (*χ* ^2^ = 6.17; *P* = 0.01), presence of sepsis (*χ* ^2^ = 7.47; *P* = 0.006), multiple blood transfusions (*χ* ^2^ = 5.11; *P* = 0.02), and delivery by caesarian section (*χ* ^2^ = 4.22; *P* = 0.04) were significantly associated with development of ROP. There were no significant differences with gender, apneic spells, jaundice, or phototherapy. *Conclusions and Relevance*. All live infants with ROP were noted to regress spontaneously in this study. Though it may not be cost effective to acquire treatment facilities at the moment (the only child with treatable disease died), facilities for screening preterm infants displaying high risk features may be essential as smaller babies are saved.

## 1. Introduction

The control of blindness in children is considered a high priority within the World Health Organization's (WHO's) VISION 2020—The Right to Sight programme [1]. Among the causes of blindness in children worldwide is retinopathy of prematurity (ROP). It is a disorder of the developing retinal vessels seen in premature infants, especially those weighing less than 1500 g or younger than 32-week gestational age at birth [2] that may or may not have received oxygen therapy in the neonatal unit [3]. It occurs from interruption of the normal progression of vessels to the peripheral retina as a consequence of premature birth with the resultant high postnatal levels of vascular endothelial growth factor (VEGF) from hypoxia leading to disorganized growth of new retinal blood vessels and increased vascular permeability [4]. ROP is classified by the lowest zones and the highest stages observed in each eye with zones depicting how far the retinal blood vessels have grown and stages depicting the severity of the disease based on the ophthalmoscopic findings at the junction between the vascularized and avascular retina [5].

ROP has a variable course with some eyes showing a mild condition that resolves completely, or it could progress to a vision threatening condition called high risk prethreshold or Type 1 prethreshold ROP. Some eyes show a more aggressive course (“Rush” disease or the newer term aggressive posterior retinopathy of prematurity-(AP-ROP)) with neovascularization forming loops and arteriovenous shunts in Retinal zones 1 or 2, that often rapidly progresses to retinal detachment and blindness. ROP is one of the important causes of blindness in children in America [2, 6] and South Africa [7, 8] and has also been known to increase the risk of other eye conditions like strabismus, glaucoma, cataracts, and myopia later in life [2, 9, 10]. As smaller and younger babies are surviving from use of modern technology of neonatal life support, the incidence of ROP has increased [11, 12].

Apart from prematurity/low birth weight, [2–8] and exposure to supplemental oxygen therapy, [2–4, 11] other implicated risk factors for ROP include hypoxia, sepsis, hypercapnia, anemia, metabolic acidosis, apneic spells, cyanotic congenital heart disease, hyaline membrane disease, multiple births, exchange blood transfusion, intraventricular haemorrhage, Vitamin E deficiency, drugs like aminophylline, and bright UV light therapy [2, 11, 13]. Genetic differences between infants may be the reason some progress despite rigorous and timely intervention, while, other infants with similar clinical characteristics, ROP regress [14].

**Figure 1:**
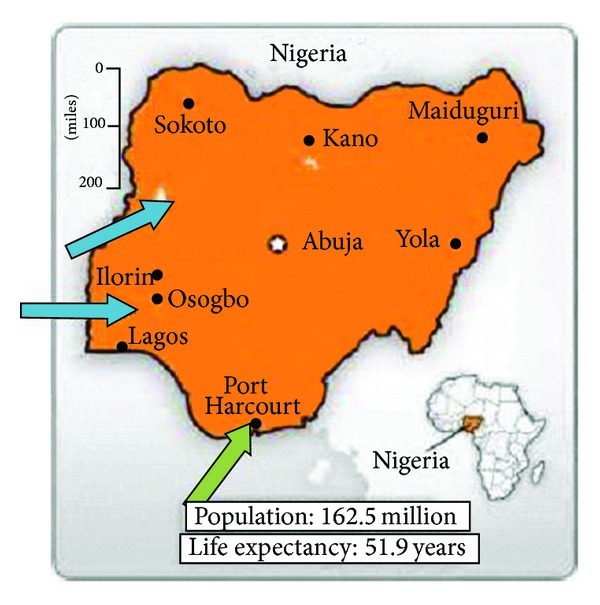
Map of Nigeria: previous studies [15, 16] (blue); present study (green).

Although ROP is important, its prevalence and hence significant contribution to causes of blindness in children in Nigeria are yet to be determined. A blind school study in north east Nigeria recorded an incidence of 0.5%, [15] while in south west Nigeria, the reported incidence of ROP in babies less than 1,500 g birth weight or less than 31 weeks gestational age was 5.5% [16] see Figure 1. Although the number of infants blinded by ROP is relatively small, this represents a great number of years of disability, which in many cases is unnecessary given that timely treatment can prevent the visual loss in these babies. Early identification of ROP by screening has therefore been recommended as a standard practice globally [17].

This study aimed at establishing the incidence and associated risk factors of ROP in Port Harcourt, Rivers State, Nigeria, and establishing a screening protocol which will identify babies with ROP in order to advise policy making bodies appropriately.

## 2. Materials and Methods

This prospective study, approved by the ethics committee of the University of Port Harcourt Teaching Hospital was carried out at the Special Care Baby Unit (SCBU) and the outpatient pediatric clinic of the University of Port Harcourt Teaching Hospital (UPTH), Port Harcourt, Nigeria, between January 1 and October 31, 2012 (a 10-month period). Since the unit started over 10 years ago, ROP screening started for the first time, just 3 months prior to start of this study. All consecutive premature neonates born before or at 32-week gestation and with a birth weight equal to or less than 1500 g admitted into the SCBU or seen upon discharge on an outpatient basis were included in this study following informed consent from parents. Neonates with congenital abnormalities of the eyes or who died before the first ophthalmologic examination were excluded.

On admission, neonatologists examined the babies and managed any existing medical conditions. The gestational age (GA) was calculated from the first day of last menstrual period (LMP) of the mother. Where this was not available, the Dubowitz score [18] was used to determine the gestational age. The babies were weighed at birth naked with a basinet to the nearest 50 g. Other demographic information obtained and entered in the study questionnaire included the gender, mode of delivery, and reasons for prematurity in addition to Apgar scores at 1 and 5 minutes, use and duration of supplemental oxygen, presence of apneic spells, and interventions like phototherapy, exchange blood transfusion, and so forth.

### 2.1. Management of Infants at Risk of ROP

The babies were nursed in incubators until they achieve a weight of 1600 g. At all times during SCBU care, no eye shields were used except when infants were receiving phototherapy.

Supplemental oxygen was given if required to all babies who required it regardless of presence of ROP to maintain steady blood oxygenation (measured by pulse oximetry units) between 90 to 95%.

Broad spectrum antibiotics such as Ceftazidime and Gentamicin antibiotics were commenced empirically in babies with suspected sepsis, unless the result of the blood culture showed a different sensitivity pattern. Exchange blood transfusions were performed for severe neonatal jaundice or severe anemia. None of the infants had cranial ultrasonography carried out or surfactants administered due to nonavailability.

### 2.2. Eye Examination Schedule

All the eye examinations of the babies were conducted by a single ophthalmologist at 4 weeks postnatally or at 33-week postmenstrual age (gestational age at birth plus chronological or postnatal age) whichever was later in a detailed manner.

### 2.3. Eye Examination Method and Monitoring

Pupillary dilatation was achieved using 2 drops of 1% Tropicamide and 2.5% Phenylephrine combination at 5 minutes interval, with the excess wiped away, followed by punctual occlusion. The eye examination was conducted usually within 45 minutes. Topical anesthetic (1% Proparacaine) was used and the SCBU or clinic room lights were dimmed. The examination protocol included examining the fundus using an indirect ophthalmoscope (Welch Allyn Binocular Indirect Ophthalmoscope Model 12500, Welch Allyn Inc., Skaneateles Falls, NY) and a Volk 20D lens (V20LC), holding the lids gently apart with a lid speculum (Sauer Premature Infant Speculum Model K1-5302, Katena Products Inc., Denville, New Jersey, USA) and using a sclera depressor to see the extreme periphery (Flynn Scleral Depressor, Model E5107, (Bausch + Lomb Storz ophthalmics), and Volk 20D (V20LC). Topical balanced salt solution (BSS) was used to keep the cornea moist during the examination.

The retinal findings were documented carefully in examination sheets according to the international classification according to the criteria established by the International Committee for Classification of ROP (ICROP) [5, 19].

The follow-up schedule was based on initial retinal findings as per earlier published protocols [20].

Babies identified to have vision threatening ROP (ETROP guidelines) [21] were referred to another center about 800 miles away outside the state where facilities were available since treatment facility was not available in our center.

All external and anterior segment examination findings were similarly documented.

#### 2.3.1. Statistical Analysis

The maximum severity of ROP in any one eye for an individual infant was used for analysis. All data was analyzed with the assistance of a statistician using Epi Info version 6.1 (Center for Disease Control & Prevention, GA). Univariate comparisons of risk factors between the 2 groups with and without ROP were evaluated using the chi-square test with statistical significance taken to be *P* < 0.05.

## 3. Results

A total of 550 neonates were admitted into SCBU within the study period and included 450 (81.8%) term babies and 100 (18.2%) preterm babies. Of the 100 preterm babies, 87 survived. Fifty-three of these met the inclusion criteria and were enrolled into the study: thirty-one (58.5%) males and twenty-two (41.5%) females, with ratio of 1.4 : 1. Mean gestational age at birth was 28.98 ± 1.38 weeks. (range 26 to 31 weeks). Mean birth weight was 1411 ± 128 g (range 900 g to 1500 g) and mean Apgar score at 1 minute was 5.1 ± 1.4 and at 5 minutes the mean was 7.2 ± 1.1.

Premature rupture of membranes in 22 (41.5%) and premature onset of labor in 13 (24.5%) were the commonest reasons for prematurity (Table 1). Twenty-two of them (41.5%) were products of multiple gestations. Twenty-six (49.1%) were delivered vaginally while 27 (50.9%) were by caesarian section. Two (3.8%) were albinotic babies.

### 3.1. Associated Systemic Conditions and Interventions

Neonatal jaundice (NNJ) was observed in 51 (96.2%), sepsis in 24 (45.3%), and birth asphyxia in 22 (41.5%) (Table 2). One (1.9%) of the babies had multisystem congenital anomalies involving the cardiac system and musculoskeletal system along with spina bifida.

Forty-nine (92.5%) babies received phototherapy, with mean duration of 10.1 ± 2.8 days (range 7–18 days). Exchange blood transfusion was performed in 44 (83%) babies with 23(52.3%) of them receiving multiple blood transfusion. Thirty-one (58.5%) received supplemental wall piped oxygen with mean duration of 3.04 ± 2.3 days (range 2–10 days). In 3 (5.7%) of the babies, the mothers received steroids to facilitate lung maturity prior to delivery.

### 3.2. The Ocular Features of the Study Population

#### 3.2.1. Anterior Segment Features

Mild to moderate conjunctivitis (ophthalmia neonatorum) was found in 19 (36%) cases. One of the babies had seborrheic blepharitis and the anterior chamber depth was shallow in 51 (96.2%) cases. One baby had poorly-dilating pupils due to neovascularization of the iris (NVI).

#### 3.2.2. Posterior Segment Features

Of the 53 eyes studied, 25 (47.2%) were diagnosed with different degrees of ROP.  Table 3 shows the prevalence according to birth weight and gestational age stratification. The prevalence among babies weighing ≤1300 g was 75% and among those delivered before 30 completed weeks was 58%.

One (4%) had disease in Zone I, 5 (20%) in Zone II, and 19 (76%) in Zone III (Table 4). The rest, 28 (52.8%), had mature retina extending to Zone III. The one patient with disease in Zone I had stage 3 disease involving at least 6 contiguous hours in addition to plus disease and difficulty in pupillary dilatation due to iris neovascularization. This was the patient with multiple congenital anomalies (outside of the eye) and who was classified as having threshold disease and qualified for therapy, but he died within 20 days of birth before he could be referred. In total, twenty-one (84%) had stage 1 no plus disease while 3 (12%) had stage 2 no plus disease. None had stage 4 or stage 5 disease or AP-ROP.

Table 5 compares some risk factors between preterm babies with ROP and preterm babies without evidence of ROP.

### 3.3. Follow-Up

Nineteen eyes (79%) (except the one case with threshold disease that died after only one session of eye examination) had regressed completely by 38 weeks after menstrual age (PMA) while all 24 (100%) had regressed by 42 weeks PMA.

## 4. Discussion

From 1942 when Terry [22] described ROP (which he called retrolental fibroplasia), it has continued to be a significant complication in preterm neonates and an important cause of potentially preventable blindness worldwide. Phelps in 1979 [23] implicated prematurity as an etiology of ROP.

The prevalence of ROP in our study was 47.2%. This was higher than the 19.2% reported by Hakeem et al. [24] and the 10.8% in Beijing [25]. This may be because these studies included infants with higher gestational age (>32 weeks) and higher birth weight (up to 2 kg). The incidence of ROP is known to be higher with decreasing gestational age and birth weight. The prevalence in our study was also higher than that reported in developed countries [26–28]. In developed and industrialized countries where enough financial resources allow for provision of optimum care of extremely immature newborns, adequate screening, and treatment, the rates of blindness from ROP have declined [26–28]. In contrast, in less developed countries, due to limited financing resources, the increasing survival of premature newborns is not matched by high levels of standard of care, thus resulting in increasing prevalence or the so-called third ROP epidemic [10].

The frequency and degree of the disease are inversely related to the gestational age and weight at birth. Similar was the finding in our study where the prevalence in babies weighing less than 1300 g was 75% and 58% in babies delivered before 30 weeks. Vyas et al. [29] reported an incidence of 47% in infants with birth weights between 1000 and 1251 g and 81.6% for infants weighing less than 1000 g at birth, while only 60% of infants born at 28–31 weeks developed ROP and over 80% of infants born at less than 28-week gestational age developed ROP.

Other implicated risk factors are hypoxia and receiving supplemental oxygen [3, 22]. In our study also significantly more children who received supplemental oxygen developed ROP. Oxygen therapy by itself and also fluctuation in its levels have been implicated in the rapid progression of ROP in these infants and therefore constant monitoring to within levels less than 90% in their management with the minimum of pulse oximeters. Our monitoring however is up to standard protocols but still requires to be improved upon.

Sepsis was significantly associated with development of any degree of ROP. This was also reported by other studies [24, 30]. In sepsis, microorganisms infiltrate vascular endothelial cells of the eyes and induce phagocytosis, endothelial cell damage, and release of proinflammatory cytokines especially endothelial growth factor which has been specifically implicated in the pathogenesis of ROP [31]. The debilitation it produces also has deleterious effect on the children inhibiting rapid weight gain the absence of which is another factor implicated in the progression of ROP. Babies that received multiple blood transfusions also developed ROP which has been identified as a risk factor for ROP in several studies [26, 32, 33]. Damaging effects on the retina through increase in free iron that may catalyze Fenton reactions, to produce free hydroxyl radicals capable of damaging the retina [32–34].

Significantly more babies delivered by caesarian section had ROP as also reported by Shah et al. [35]. While one study in contrast reported that vaginal delivery was a significant and independent predictor of threshold ROP in ELBW infants, [36] others found no relationship with the mode of delivery [26, 37] Gender and receiving phototherapy were not identified as risk factors in our study as was also reported by others [26]. In contrast, Darlow et al. [38] found the male gender a significant risk factor to development of ROP.

As reported in earlier ROP studies, [5, 39, 40] spontaneous resolution is seen in most (80%) cases without visual loss from retinal detachment or scars. In our study, this could be because very sick and vulnerable babies may not have survived.

However, these babies will still require periodic followup despite regression as the risk of other eye conditions like myopia and strabismus is known to be increased in them [2, 9, 10].

## 5. Conclusion 

Though the prevalence of ROP in this cohort of preterm babies is high (47.2%) it did not progress to severe blinding disease; rather it spontaneously regressed and therefore the problem is actually very low. However screening identified at least one vision threatening ROP in our study. Better survival in the coming years could lead to increased epidemic of ROP blindness. Though investing in equipment for treatment may not be completely necessary at this point of time, it is important to set up screening protocols and its attendant equipment in our SCBUs to be able to identify the few who may develop vision threatening disease. Babies do not become blind as numbers but each baby becomes blind as an individual.

## Figures and Tables

**Table 1:** Reasons for premature delivery.

Reasons for prematurity	No. (%)
Premature rupture of membrane	22 (41.5%)
Premature labour	13 (24.5%)
Preeclamptic toxaemia and eclampsia	8 (15.1%)
Placenta praevia	6 (11.3%)
Precious baby	3 (5.7%)
Cephalopelvic disproportion	1 (1.9%)

Total	53 (100%)

**Table 2:** Comorbidities and interventions in the 53 preterm babies in the study group.

Co-morbidities/interventions	No (%)
Co-morbidities	
Neonatal jaundice	51 (96.2)
Sepsis	24 (45.3)
Birth asphyxia	22 (41.5)
Ophthalmia neonatorum	19 (35.8)
Anemia	17 (32.1)
Congenital pneumonia	14 (26.4)
Apnoea	13 (24.5)
Hypoglycemia	8 (15.1)
Renal failure	6 (11.3)
G6PD deficiency	3 (5.7)
Patent ductus arteriosus	3 (5.7)
Multisystem congenital anomalies	1 (1.9)
Interventions	
Phototherapy	49 (92.5)
^¶^Exchange blood transfusion	44 (83.0)
Supplemental oxygen	31 (58.5)
Mechanical ventilation	0 (0.0)
Surfactant	0 (0.0)

^¶^Twenty-three had multiple blood transfusions.

**Table 3:** Prevalence of ROP according to birth weight and gestational age.

Characteristics	With ROP	Without ROP	Total
Birth weight < 1300 g	6 (75%)	2 (25%)	8 (100%)
Birth weight ≥ 1300 g	19 (42%)	26 (58%)	45 (100%)
Gestational age < 30 weeks	18 (58%)	13 (42%)	31 (100%)
Gestational a ge ≥ 30 weeks	7 (32%)	15 (68%)	22 (100%)

**Table 4:** Stages of ROP in 25 preterm babies.

Zones	Stage 1	Stage 2	Stage 3	Total
Zone I	0 (0%)	0 (0%)	1 (4%)	1 (4%)
Zone II	3 (12%)	2 (8%)	0 (%)	5 (20%)
Zone III	18 (72%)	1 (4%)	0 (0%)	19(76%)

Total	21 (84%)	3 (12%)	1(4% )	25 (100%)

**Table 5:** Comparison of some risk factors between preterm babies with ROP (*n* = 25) and preterms without evidence of ROP (*n* = 28).

Risk factors	With ROP	Without ROP	*χ* ^2^ value	*P* value
Mean gestational age (weeks)	28.9	29.8	0.00	1.0
Mean birth weight (g)	1372	1446	0.01	0.94
Use of supplemental oxygen	24	7	6.17	0.01*
Presence of sepsis	20	4	7.47	0.006*
Apnoeic spells	9	4	1.25	0.26
Phototherapy	25	24	0.04	0.8
Neonatal jaundice	25	26	0.0	1.0
Multiple blood transfusion	18	5	5.11	0.02*
Male gender	19	12	1.05	0.3
Female gender	6	16	1.78	0.18
Vaginal delivery	5	21	4.66	0.03*
Caesarian section	20	7	4.22	0.04*

(*P ≤ 0.05).
